# A novel and ancient group of type I keratins with members in bichir, sturgeon and gar

**DOI:** 10.1186/1742-9994-4-16

**Published:** 2007-06-06

**Authors:** Michael Schaffeld, Mark Haberkamp, Sonja Schätzlein, Sebastian Neumann, Christian Hunzinger

**Affiliations:** 1 Institute of Zoology, Johannes-von-Müller-Weg 6, Johannes Gutenberg University, D-55099 Mainz, Germany; 2 Dept. of Gastroenterology, Medical School Hannover, Carl Neuberg Str. 1, K11, E01, R1400, 30629 Hannover, Germany; 3 Merck KGaA, Central Services Analytics, Central Product Analytics/Bioanalytics, Frankfurter Str. 250, D-64293 Darmstadt, Germany

## Abstract

**1. Background:**

Vertebrate epithelial cells typically express a specific set of keratins. In teleosts, keratins are also present in a variety of mesenchymal cells, which usually express vimentin. Significantly, our previous studies revealed that virtually all known teleost keratins evolved independently from those present in terrestrial vertebrates. To further elucidate the evolutionary scenario that led to the large variety of keratins and their complex expression patterns in present day teleosts, we have investigated their presence in bichir, sturgeon and gar.

**2. Results:**

We have discovered a novel group of type I keratins with members in all three of these ancient ray-finned fish, but apparently no counterparts are present in any other vertebrate class so far investigated, including the modern teleost fish. From sturgeon and gar we sequenced one and from bichir two members of this novel keratin group. By complementary keratin blot-binding assays and peptide mass fingerprinting using MALDI-TOF mass spectrometry, in sturgeon we were able to assign the sequence to a prominent protein spot, present exclusively in a two-dimensionally separated cytoskeletal preparation of skin, thus identifying it as an epidermally expressed type I keratin. In contrast to the other keratins we have so far sequenced from bichir, sturgeon and gar, these new sequences occupy a rather basal position within the phylogenetic tree of type I keratins, in a close vicinity to the keratins we previously cloned from river lamprey.

**3. Conclusion:**

Thus, this new K14 group seem to belong to a very ancient keratin branch, whose functional role has still to be further elucidated. Furthermore, the exclusive presence of this keratin group in bichir, sturgeon and gar points to the close phylogenetic relationship of these ray- finned fish, an issue still under debate among taxonomists.

## Background

In vertebrates the cytoskeleton of epithelial cell types is typically reinforced by a specific set of type I and type II keratins that assemble into 10 nm thick filaments formed from typeI/II heterodimers. The keratins are members of the large multigene family of intermediate filament proteins (IFproteins) of which they form by far the most complex group. In human, 53 of the hitherto nearly 70 identified IF protein genes code for keratins [1-4] that are expressed in tissue and developmental specific patterns. Without including human hair and nail forming keratins, the number of keratin genes found in teleost fish is comparably high, but in contrast to human and other tetrapods, teleost fish possess a large excess of type I keratin genes [5-7]. By analysing the molecular evolution of keratins, as well as the evolution of their expression patterns in lower vertebrates, we want to further elucidate the scenario and probable evolutionary forces that led to this extraordinary variety of keratins in vertebrates.

Our investigations of the keratin systems in lamprey, shark, trout, zebrafish, carp, goldfish and lungfish have so far revealed that type I and type II keratins are apparently present in all classes of vertebrates and that the various keratins can generally be subdivided into the "E" keratins, expressed in epidermal keratinocytes and other stratified epithelia, and those appearing in cells forming simple epithelia, thus named "S" keratins [8-19]. Nevertheless, our data based on cDNA sequence analysis, followed by thorough phylogenetic analyses [13-18] as well as the studies based on the recently available genome data from man and teleost fish [1,2,5-7], strongly support the view of largely independent origins of the keratin genes found in fish and man. According to our present data, solely the typical "S" keratin pair K8 and K18 can at least be found in all gnathostomian vertebrate groups, indicating the unique and general importance of these "ancient" keratins. In contrast to other vertebrates investigated so far, in modern teleost fish keratins, including K8 and K18, in addition to their typical IF epithelial appearance show a widespread IF occurrence in mesenchymally derived cells and tissues, such as fibroblasts, chondrocytes and blood vessel endothelia (for review see [11]). The latter in the non-teleost vertebrates usually do not express keratins but the type III IF protein vimentin [20-26]. To further trace the origin of the different "E" and "S" keratins as well as the evolution of the mesenchymal keratin expression in teleosts, we have investigated the keratin systems in a bichir, a sturgeon and a gar that are believed to represent the most ancient groups of the extant ray-finned fish. In the course of these studies, from all three species we obtained sequences that apparently belong to a novel branch of type I keratins, without counterparts in any other vertebrate group investigated so far, including the teleost fish. Here we present and discuss their sequences as well as their phylogenetically relationships to the other members of the type I keratin subfamily, which may also provide clues to the early evolution of ray-finned fish. The latter is still strongly debated among taxonomists, whether on the basis of molecular or morphological data (for an overview see [27]).

## Results and discussion

### Novel type I keratin sequences from bichir, sturgeon and gar

**Figure 1:**
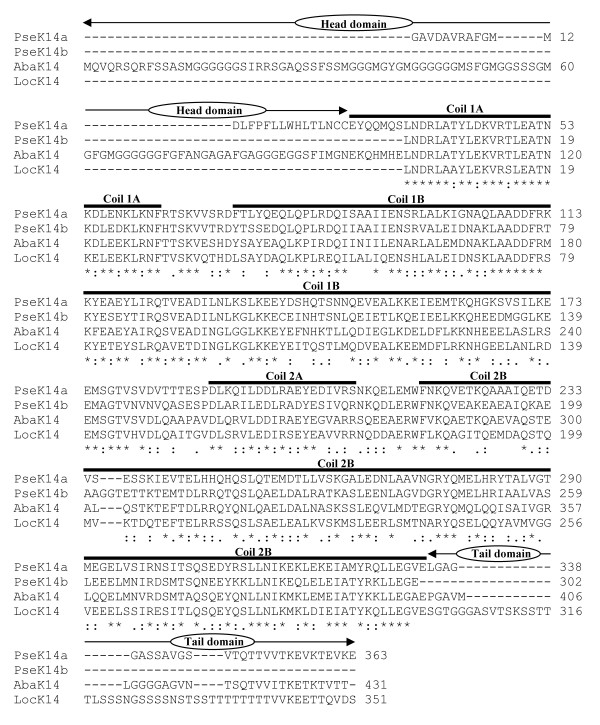
**Sequence comparison of K14 from bichir, sturgeon and gar**. Multiple alignment of the keratin 14 (K14) sequences we obtained from bichir, sturgeon and gar. Thick black lines mark the four helical subdomains (coils 1A to 2B), which are typical for the central rod domain of all known IF-proteins. Asterisks indicate identical amino acids; double dots indicate a high and single dots a lower degree of amino acid conservation. Pse, *Polypterus senegalus *(bichir); Aba, *Acipenser baeri *(sturgeon); Loc, *Lepisosteus oculatus *(gar). Note that only AbaK14 comprises the complete amino acid sequence. From PseK14a at least a section and from LocK14 the complete head sequence is still missing. From PseK14b we still have to recover both, the complete head and tail sequence.

Only recently we have discovered that a rather ancient and distant group of keratin-related sequences, the extracellularly secreted thread keratins TKα and TKγ, are not only present in hagfish (the assumedly most ancient vertebrate group), but also in lamprey, teleosts and amphibians. This provided major clues relating to keratin evolution in vertebrates, but also pointed to a more general role of this previously considered highly specific IF protein group in vertebrates [28]. By combination of RT-PCR experiments and cDNA library screening (for details see Methods), from sturgeon and gar we have now isolated one and from bichir two cDNA sequences that, according to our phylogenetic analysis, code for members of another novel keratin group (Fig. 1, Table 1). The 1858 bp long cDNA clone we isolated from the sturgeon cDNA library (*abak14*; [EMBL: AJ493259]) contains the complete coding sequence for a type I keratin of 46759Da (431 amino acids) and a calculated pI of 5.1, which we now term AbaK14 (from *Acipenser baeri *keratin). However, the corresponding sequences we have so far obtained from bichir and gar are still incomplete. A 1369 bp long clone (*pseK14a*; [EMBL: AM419452]) isolated from the bichir cDNA library encodes a type I keratin that we term PseK14a (*Polypterus senegalus *keratin). It still lacks a portion of its head encoding sequence in addition to its 5' UTR. By RT-PCR using degenerate primers, from bichir we additionally recovered a 908 bp long cDNA sequence (*pseK14b*; [EMBL: AM419453]), comprising almost the complete rod encoding segment of a second K14 counterpart in this species (PseK14b). In addition, we found five further incomplete cDNA clones that apparently encode different variants of PseK14a (not shown here). The latter only slightly vary in DNA sequence, from 0.1 – 3.1% (amino acid variance of 0.4 – 4.9%). In a similar way we were also able to amplify a cDNA fragment encoding the rod domain of a K14 counterpart in gar, which we term LocK14 (from *Lepisosteus oculatus *keratin). By RACE-PCR we additionally recovered its tail encoding sequence and 3' UTR. Its assembled sequence (*lock14*; [EMBL: AM419454]) overall comprises 1207 bp, but still lacks the complete head encoding segment in addition to the section coding for the first seven residues of the rod domain.

**Table 1:** Properties of the isolated cDNA clones encoding K14 from bichir, sturgeon and gar.

**cDNA clone**	**Keratin**	**EMBL accession number**	**Size of cDNA (bp)**	**Number of encoded amino acids**	**Mr (Da)**	**pI**
*abaK14*	AbaK14	AJ493259	1858	431	46759^4)^	5.1^4)^
*pseK14a*	PseK14a	AM419452	1369	363^1)^	-	-
*pseK14b*	PseK14b	AM419453	908	302^2)^	-	-
*locK14*	LocK14	AM419454	1207	351^3)^	-	-

Pse, *Polypterus senegalus *(bichir); Aba, *Acipenser baeri *(sturgeon); Loc, *Lepisosteus oculatus *(gar); ^1) ^a portion of the head domain is still missing; ^2) ^head and tail domain are still missing; ^3) ^complete head domain is still missing; ^4) ^as predicted from the cDNA sequence.

Subsequent mining of the available genome and EST databases for K14 counterparts in other vertebrates such as teleosts, amphibians, birds and mammals, so far has not yet yielded any matches, suggesting that this keratin group may only be present in the ancient groups of ray- finned fish. We only found two matches encoding K14 of another sturgeon, notably *Acipenser transmontanus *(white sturgeon; [EMBL: DR975435, DR975694]), which both stem from a skin-derived cDNA library.

### Biochemical identification of K14 in sturgeon

**Figure 2:**
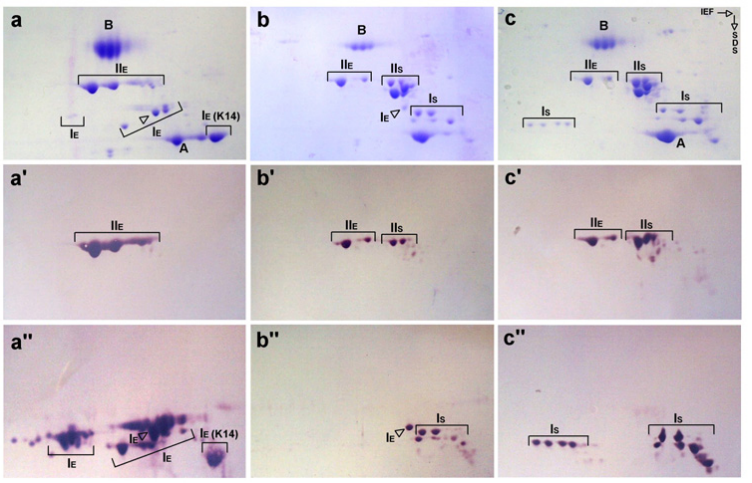
**Biochemical identification of K14 in sturgeon**. 2D-PAGE of cytoskeletal proteins extracted from sturgeon skin (a), stomach (b) and intestine (c). Isoelectric focusing (IEF) was used in the first dimension, in the second dimension we applied SDS-PAGE. Bovine serum albumin (B) and rabbit α-actin (A) were added to the samples as marker proteins. E, epidermal keratins; S, simple keratins. (a-c) Coomassie Blue stained gels, showing the polypeptide patterns, which were subsequently used for complementary keratin blot-binding (CKBB) assays. (a'-c') CKBB test employing biotinylated human keratin K18, thereby identifying type II keratins (II). (a"-c") CKBB assay using biotinylated human keratin K8 which identifies type I keratins (I). Note that the K14 spot is not present as a major component in intestine or stomach and that both tissues express a mixture of E and S keratins.

To analyse the general set of keratins expressed in sturgeon, we extracted the cytoskeletal proteins from different tissues, including skin, liver, intestine, stomach and gill, separated them by 2D-PAGE and subsequently analysed the patterns by CKBB assays and immunoblotting (results for skin, stomach and intestine are shown in Fig. 2). The major spots were additionally analysed by peptide mass fingerprinting (PMF) to reveal similarities between identified keratin spots and to assign them to the sequences we obtained from the sturgeon by cDNA library screening. In the course of these investigations we were clearly able to assign a single protein spot to the amino acid sequence derived from the cloned *abak14 *cDNA sequence. The matching spot was solely found as a major component in the cytoskeletal preparation of skin (Fig. 2a), in which it was firmly identified as a type I keratin in the CKBB assay (Fig. 2a"). It showed a positive reaction with the anti-trout-keratin antiserum GPpoly (data not shown), which we previously introduced as a general keratin marker in fish [8,10-15,19]. The two-dimensional position of this protein spot fits the theoretical values calculated from the sequence of AbaK14 (see above and Table 1). Peptide mass fingerprint (PMF) analysis of this protein yielded 29 matching peptide masses for AbaK14, of which 26 were specific for AbaK14 in comparison to the other six type I keratins we so far sequenced from sturgeon. Overall amino acid sequence coverage for AbaK14 was 61%.

### Molecular evolution of keratins – the ancient origin of K14

**Figure 3:**
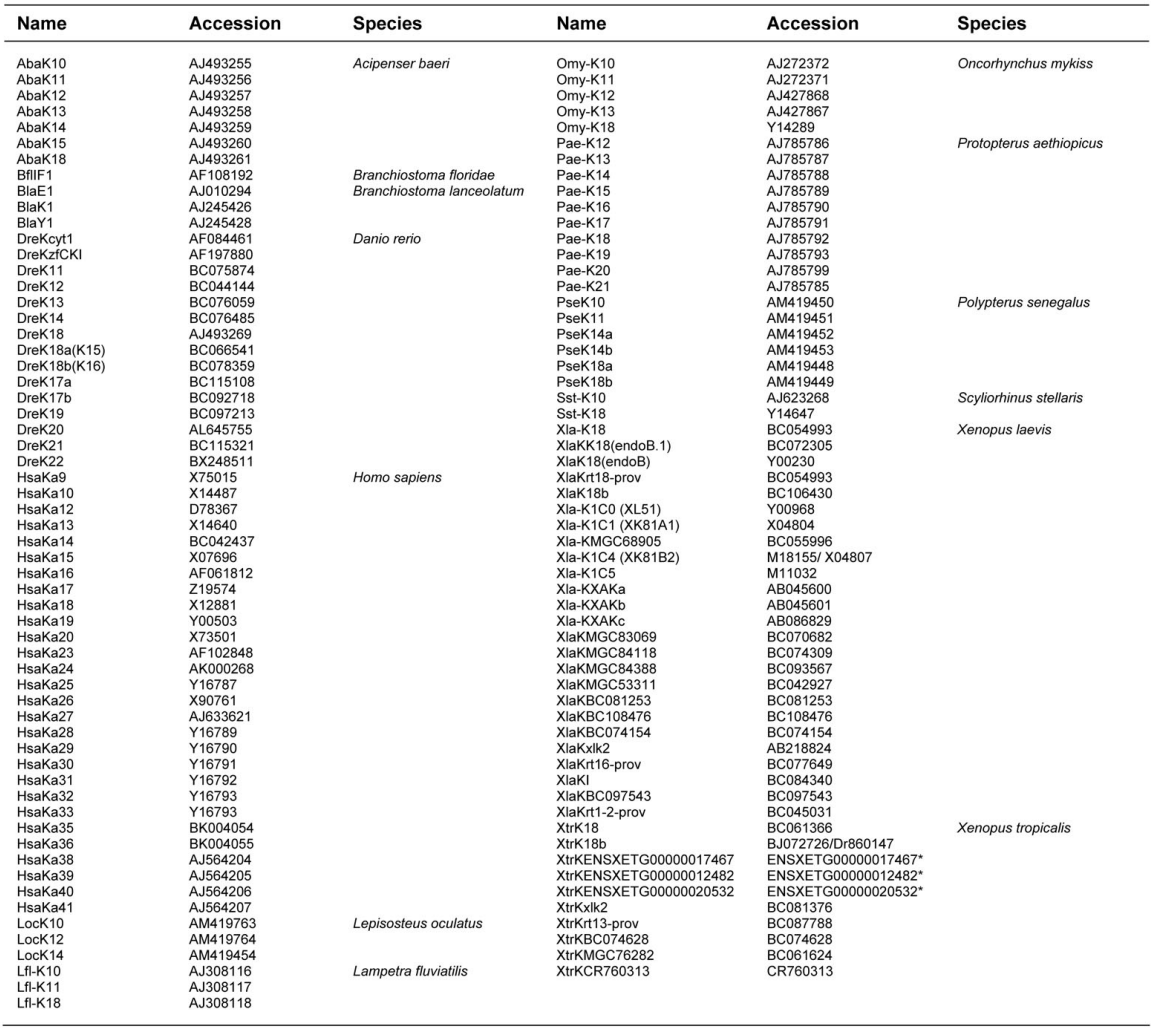
**Accession numbers**. EMBL accession numbers of the type I keratin sequences we used for phylogenetic inference. *For three *Xenopus tropicalis *type I keratin sequences the Ensembl database gene IDs are given.

**Figure 4:**
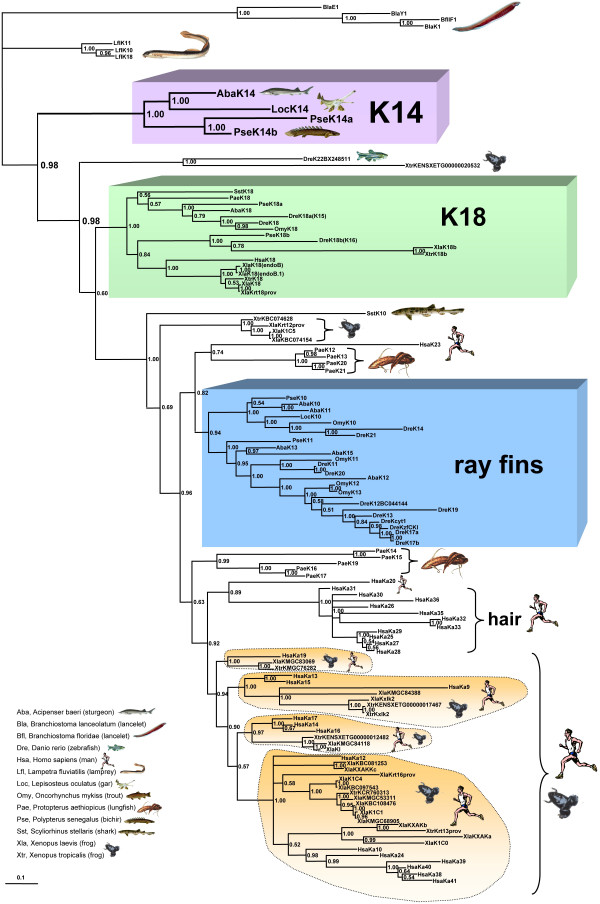
**Molecular evolution of type I keratins**. Phylogenetic tree based on Bayesian inference, illustrating the relationships of the K14 sequences from bichir, sturgeon and gar to the other type I keratins known from vertebrates. The tree was rooted with the lancelet type I keratin sequences. It clearly shows that the K14 sequences form a separate branch (boxed in violet) close to the sequences we cloned from the river lamprey. They even branch off prior to the twig formed by the gnathostomian K18 sequences (boxed in green) that apparently emerged before the separation of cartilaginous and bony fish. The tree, furthermore, suggests that most of the ray-finned fish type I keratins (boxed in blue) evolved independently from those present in lungfish, frog or man and that early in actinopterygian evolution gene duplications already gave rise to at least two different type I keratin groups with members in both, ancient and modern ray-finned fish. Importantly, within the tetrapod lineage the Bayesian analysis revealed four highly supported keratin subgroups, each with members in both, frog and man (encircled by dotted lines and coloured orange). Bar, 0.1 substitutions per site.

To infer the phylogenetic relationships of the novel K14 group to the other currently known type I keratins, by different methods we thoroughly analysed a comprehensive data set of 118 polypeptides, including the type I keratins from lancelet, lamprey, shark, bichir, sturgeon, gar, zebrafish, trout, *Xenopus*, lungfish and man (for further details see Methods; the available accession numbers of the employed sequences are listed in Fig. 3). Most of the fish sequences stem from our own data, notably those from lamprey, shark, bichir, sturgeon, gar, trout and lungfish in addition to K8 and K18 from zebrafish [13-18,23,29,30]. We rooted the trees with the type I keratin sequences available from the lancelets, which are believed to represent the most ancient group of living chordates [31]. In general we received the same basal tree topology when applying Neighbor Joining (NJ), Maximum Likelihood (ML) or Bayesian (B) methods for our phylogenetic analysis. The phylogenetic tree shown in Fig. 4 is based on Bayesian inference and clearly shows that the K14 sequences form a separate and basal branch within the type I keratins, phylogenetically close to the sequences we obtained from the river lamprey. They even branch off prior to the twig formed by the gnathostomian K18 sequences, that apparently emerged before the separation of cartilaginous and bony fish [13-15]. This ancestral origin of the K14 twig together with the assumedly exclusive presence of K14 in ancient ray-finned fish (see above) raises the question as to the complementary binding partner(s) of this keratin group. So far, analysis of bichir and sturgeon has not revealed a group of type II keratins occupying a similar basal position equivalent to the K14 sequences in the type I keratin tree [8,29,30]. Moreover, the suggested restriction of K14 sequences to bichir, sturgeon and gar indicates the close phylogenetic relationship of these fish groups, an issue still under debate among taxonomists. Based on recent molecular data it can be concluded that sturgeons, gars and bowfin together may form a sister group to the teleost fish and that bichirs represent the most ancient group of ray-finned fish (for review see [27]). Since our current studies do not contribute to the resolution of this problem, the keratin system in bowfin should be investigated, which might shed more light on this issue.

Furthermore, the phylogenetic tree illustrated in Fig. 4 suggests that most of the type I keratins from ray-finned fish evolved independently from those present in lungfish, frog or man and that very early in actinopterygian evolution a gene duplication gave rise to at least two different type I keratin branches, each with members in both ancient and modern ray- finned fish (see the boxed ray-fins twig in Fig. 4). Compared to our recent analyses [14-18,28], we have now included additional type I keratin sequences from zebrafish and *Xenopus*; therefore, in this study the K18 branch shows a more complex branching pattern, not allowing a clear identification of the authentic K18 counterparts in the different vertebrates solely on the basis of their position in the tree. However, our previous identification of K18 in shark, bichir, sturgeon, trout and zebrafish was additionally based on its typical occurrence in simple epithelia, such as liver hepatocytes or intestinal mucosal epithelium, corresponding to the situation in man and other tetrapods [10,12-16,19,29]. Nevertheless, these "functional K18 counterparts" identified in the different vertebrate groups have to be considered as paralogous protein sequences. The branching pattern of the K18 twig supports the suggestion that early as well as more recent duplication events led to the various K18 related genes. Future analysis of their expression patterns may provide further clues for possible functions of these K18 relatives.

Compared to the evolution of type II keratins, the phylogenetic tree of type I keratins appears rather complex and several nodes cannot be resolved. Moreover, in teleosts the number of detected type I keratin genes virtually triples the number of those coding for type II keratins [5-7], in contrast to the almost equal number of type I and type II keratin genes detected in the tetrapod genomes. However, when including the hair- and nail-forming keratins the total number of keratin genes in tetrapods is clearly higher than in teleost fish [1-4]. A group of two sequences, one from zebrafish, the other from *Xenopus*, also occupy a rather basal position in the type I keratin tree, directly branching off between the K14 and K18 twig. But it has to be taken into consideration that both sequences stem from genomic DNA sequencing and may only represent non-active "relics" of ancient keratin genes. From the current Bayesian phylogenetic analysis (but not from our NJ and ML analysis) another phenomenon emerged for the first time: There are now four highly supported keratin subgroups in the tetrapod twig, each with members in both, frog and man (in Fig. 4 encircled by dotted lines). Moreover, the tree topology indicates that the human hair keratins may have emerged prior to these radiation events.

## Conclusion

The type I and type II keratins represent the two most complex and abundant groups of intermediate filament (IF) proteins among vertebrates and their structure, function and extraordinary variety cannot be completely understood without phylogenetic considerations. Therefore, we investigated the keratin systems in more ancient representatives of the vertebrate lineage. In particular, the keratins present in skin may play an important role in the transition of vertebrates from water- to land- living animals. Here we present a novel group of epidermal type I keratins, which we termed keratin 14 and so far have only found them in the basal groups of living ray-finned fish, notably bichir, sturgeon and gar. Our phylogenetic analysis revealed a rather basal position of this keratin group in the tree of type I keratin evolution. The keratin 14 group even emerged prior to the gnathostomian K18 sequences, which together with its binding partner K8 and with the exception of the recently discovered thread keratins in teleosts and amphibians, was hitherto considered as most ancient group of gnathostomian keratins. Future analyses will hopefully shed more light on the expression and functional role of K14 in the fish epidermis and clarify the identity of its type II keratin binding partner.

## Methods

### Preparation of tissues and cytoskeletal proteins

Sturgeons (*Acipenser baeri*) were purchased from a local hatching farm (Fischzucht Rhönforelle, Gersfeld, Germany), and gars (*Lepisosteus oculatus*) from a local pet shop (Fauna Exotica, Mainz-Kostheim, Germany). The bichirs (*Polypterus senegalus*) were a gift from Dr. Latz (Institute of Zoology, University of Mainz). Within the scope of this study we employed one specimen for each species. Animals were killed by cutting the neck or the tail artery after MS 222 narcosis (0.5 g/l). For further procedures, the tissues were excised and used immediately or snap frozen according to [8]. Cytoskeletal proteins were extracted as described in [8].

### Electrophoresis, immunoblotting, CKBB, PMF and immunofluorescence microscopy

Two-dimensional polyacrylamide gel electrophoresis (2D-PAGE), complementary keratin blot-binding (CKBB) assays, Western blotting and indirect immunofluorescence microscopy were essentially performed as described in [8]. For immunoblotting we used 10% milk powder as the blocking reagent and the antibody incubations were performed overnight at 8°C. Peptide mass fingerprinting (PMF) using matrix-assisted laserdesorption/ionization time-of-flight mass spectrometry (MALDI-TOF MS) was performed as described in [16,18]. The mass tolerance for matching fragments was set to ≤ 100 ppm.

### Preparation of RNA

Total RNA from bichir was extracted according to a protocol modified from [32], including a GTC extraction and subsequent sedimentation of RNA by ultracentrifugation through a dense cushion of caesium chloride (detailed protocol given in [8]). For purification of sturgeon RNA we applied a protocol that included consecutive steps of guanidinium thiocyanate (GTC) homogenizations, acidic phenol/chloroform extractions and precipitations. The final RNA precipitation was accomplished with 8 M LiCl. Gar RNA was prepared using the GeneMATRIX Universal RNA Purification Kit purchased from Roboklon (Germany) according to the suppliers' instructions. Isolation of mRNA was generally performed with the "PolyATract^®^-mRNA isolation system" from Promega.

### RT-PCR analyses

Applying 0.5 to 1.0 μg of total RNA, RT-PCR was performed either using the Superscript II reverse transcriptase from Invitrogen (RT for 1 h at 42°C), followed by standard PCR (Taq Polymerase, Invitrogen) or the QIAGEN^®^OneStep RT-PCR Kit (RT for 0.5 h at 50°C). PCR was done for 30–35 cycles and primer specific annealing temperatures. We used different combinations of degenerate, IF-specific primers, as previously described [8] (notably primer numbers P4, P5, P8, P9, P10). 3' RACE-PCR was accomplished using an oligodT-19mer as downstream and sequence-specific oligos as upstream primers. For gel extraction of PCR products we employed the QIAquick Gel Extraction Kit (Qiagen) or the GeneMATRIX DNA Purification Kit AGAROSE-OUT (Roboklon). Cloning of the isolated DNA fragments was accomplished either with the TOPO-TA Cloning Kit (Invitrogen), the StrataClone™ PCR Cloning Kit (Stratagene) or the pGEM-T Easy vector kit (Promega). All kits were used according to the instruction manual. Primers were synthesized by Roth and Sigma-Ark, respectively. Nucleotide sequencing was performed on both strands using the Taq Dye Deoxy Terminator system. The subsequent gel run was done by commercial services (Genterprise, Germany).

### cDNA library construction and screening

According to the supplier's instructions we constructed λ-phage cDNA libraries (ZAP-Express^®^, Stratagene) from sturgeon and bichir, respectively, in each case using 5 μg of mRNA purified from a mixture of tissues, including skin, eyes, brain and internal organs. Clones were isolated using the fish keratin-specific antiserum GPpoly [8] and digoxygenin-labeled cDNA probes derived from obtained RT-PCR fragments. Digoxygenin labeling was either performed using the DIG-High Prime Kit from Roche, according to the instruction manual or by standard PCR, using digoxygenin labeled nucleotides from Roche.

### Sequence analyses and phylogenetic inference

For database searches, we employed the Basic Local Alignment Search Tool (BLAST [33]) of the National Center for Biotechnology Information (NCBI) and of the Ensembl Genome Browser. The multiple sequence alignments were performed with ClustalX version 1.8 [34] using default gap penalties. When necessary, the alignment was edited by hand. The final alignment was analysed by Neighbor Joining (NJ), Maximum Likelihood (ML) and Bayesian methods (B). All analyses were conducted under the Jones-Taylor-Thornton substitution model of amino acid evolution (JTT [35]). Furthermore, the model applied for the ML and Bayesian analyses included observed amino acid frequencies (F), estimated proportion of invariant sites (I), and estimation of among-site rate variation for the remaining sites according to a gamma distribution (G) that was set to four rate categories. For the NJ analysis we used the programs PROTDIST and NEIGHBOR of the Phylogeny Inference Package (PHYLIP, version 3.6 b [36]). The reliability of the tree topology was then tested by bootstrap analysis [37] with 100 replications, using the PHYLIP programs seqboot, protdist, neighbor and consensus. The ML analysis was conducted with the PHYLIP-like interface PHYML [38]. For bootstrapping we generated 100 pseudo data sets. The Bayesian inference analysis was performed with MRBAYES 3.1 [39] with the frequencies fixed to the Jones frequencies. In each of the two parallel runs four Markov chains (one hot and three cold chains) were run simultaneously for 3,000,000 generations, starting with random trees. Sampling from the trees was set to every 10th generation. Under these conditions the average value for the deviation of split frequencies reached a value < 0.01. The burn-in was set to 115000, based on the stationary phase. All consensus trees were drawn using TREEVIEW version 1.6.6 [40].

## Abbreviations

CKBB Complementary keratin blot-binding

MALDI Matrix-assisted laser desorption/ionisation

TOF Time-of-flight

MS Mass spectrometry

PAGE Polyacrylamide gel electrophoresis

PMF Peptide mass fingerprint

2D Two-dimensional

## Competing interests

The author(s) declare that they have no competing interests.

## Authors' contributions

Mark Haberkamp performed the sequencing work in sturgeon and part of the biochemical work in sturgeon, including the analysis of the provided MALDI-TOF data. Sonja Schätzlein did most of the cloning experiments in bichir. All experiments were accomplished while both authors were members of our group in Mainz. Sebastian Neumann sequenced K14 from gar. Christian Hunzinger performed the peptide mass fingerprint experiments and assisted the analysis of the MALDI-TOF data while he was in the employ of ProteoSys AG, Carl-Zeiss-Str. 51, 55129 Mainz, Germany. Michael Schaffeld supervised the conception of experiments, carried out part of the molecular and biochemical studies, performed the complete phylogenetic analyses, including sequence alignment and tree reconstruction, and wrote the manuscript. All authors read and approved the final manuscript.
